# Predicting and analyzing DNA-binding domains using a systematic approach to identifying a set of informative physicochemical and biochemical properties

**DOI:** 10.1186/1471-2105-12-S1-S47

**Published:** 2011-02-15

**Authors:** Hui-Lin Huang, I-Che Lin, Yi-Fan Liou, Chia-Ta Tsai, Kai-Ti Hsu, Wen-Lin Huang, Shinn-Jang Ho, Shinn-Ying Ho

**Affiliations:** 1 Department of Biological Science and Technology, National Chiao Tung University, Hsinchu, Taiwan; 2 Institute of Bioinformatics and Systems Biology, National Chiao Tung University, Hsinchu, Taiwan; 3 Department of Multimedia Entertainment Science, Asia Pacific Institute of Creativity, Miaoli, Taiwan; 4 Department of Automation Engineering, National Formosa University, Yunlin 632, Taiwan

## Abstract

**Background:**

Existing methods of predicting DNA-binding proteins used valuable features of physicochemical properties to design support vector machine (SVM) based classifiers. Generally, selection of physicochemical properties and determination of their corresponding feature vectors rely mainly on known properties of binding mechanism and experience of designers. However, there exists a troublesome problem for designers that some different physicochemical properties have similar vectors of representing 20 amino acids and some closely related physicochemical properties have dissimilar vectors.

**Results:**

This study proposes a systematic approach (named Auto-IDPCPs) to automatically identify a set of physicochemical and biochemical properties in the AAindex database to design SVM-based classifiers for predicting and analyzing DNA-binding domains/proteins. Auto-IDPCPs consists of 1) clustering 531 amino acid indices in AAindex into 20 clusters using a fuzzy c-means algorithm, 2) utilizing an efficient genetic algorithm based optimization method IBCGA to select an informative feature set of size *m* to represent sequences, and 3) analyzing the selected features to identify related physicochemical properties which may affect the binding mechanism of DNA-binding domains/proteins. The proposed Auto-IDPCPs identified *m*=22 features of properties belonging to five clusters for predicting DNA-binding domains with a five-fold cross-validation accuracy of 87.12%, which is promising compared with the accuracy of 86.62% of the existing method PSSM-400. For predicting DNA-binding sequences, the accuracy of 75.50% was obtained using *m*=28 features, where PSSM-400 has an accuracy of 74.22%. Auto-IDPCPs and PSSM-400 have accuracies of 80.73% and 82.81%, respectively, applied to an independent test data set of DNA-binding domains. Some typical physicochemical properties discovered are hydrophobicity, secondary structure, charge, solvent accessibility, polarity, flexibility, normalized Van Der Waals volume, pK (pK-C, pK-N, pK-COOH and pK-a(RCOOH)), etc.

**Conclusions:**

The proposed approach Auto-IDPCPs would help designers to investigate informative physicochemical and biochemical properties by considering both prediction accuracy and analysis of binding mechanism simultaneously. The approach Auto-IDPCPs can be also applicable to predict and analyze other protein functions from sequences.

## Background

DNA-binding domains/proteins are functional proteins in a cell, which plays a vital role in various essential biological activities, such as DNA transcription, replication, packaging, repair and rearrangement [1]. In past, numerous computational methods predict DNA-binding domains/proteins using informative features from protein structure and amino acid sequence. The structure-based prediction methods such as [2,3] are accurate, but not capable for high throughput annotation. In this study, the sequence-based prediction methods for DNA-binding domains/proteins are investigated.

The computational methods using support vector machine (SVM) in conjunction with evolutionary information of amino acid sequence in terms of their position-specific scoring matrices (PSSMs) for predicting DNA-binding sites were successfully developed [4]. Several methods of using machine learning approaches were developed to predict DNA-binding domains/proteins from given sequences of variable lengths [5-9], shown in Table 1.

**Table 1:** Related works of predicting DNA-binding domains/proteins from sequences

Reference	Sequence type	Identity	Feature number	Representation	Feature type	Classifier
Shao et al. 2009 [8]	protein	25%	343	Seven class Conjoint triad	PCP	SVM
Fang et al. 2008 [6]	protein	35%	40	Pseudo-AA composition	PCP	SVM
Yu et al. 2006 [9]	protein	25%	132	Combined descriptors	PCP	SVM
Cai et al 2003 [5]	protein	40%	40	Pseudo-AA composition	PCP	SVM
Kumar et al. 2007 [7]	domain and protein	25%	400	PSSM	PSSM	SVM
Auto-IDPCPs	domain and protein	25%	m*	Mean value of sequence#	PCP	SVM

PCP: physicochemical property and biochemical property

*: a small number (<30) of feature vectors selected from 531 vectors

#: The averaged value of amino acids in a sequence for one property

Due to different design aims and data sets used, it is difficult to assess which feature type is the most informative cooperated with SVM by comparing with prediction accuracies only. The PSSM profile was first used with SVM to successfully predict DNA-binding domains and proteins from query amino acid sequences in the method PSSM-400 [7]. The PSSM is an effective feature type of representing DNA-binding sequences, but its ability of interpretability is not satisfactory enough in analyzing the binding mechanism. Besides PSSMs, physicochemical properties with characteristics of high interpretability were commonly used [5,6,8,9]. Some issues are concerned in designing prediction methods, described below.

1) Selection of physicochemical properties. Generally, effective physicochemical properties of amino acids are selected as prediction features by using known properties of DNA-binding mechanism and binding knowledge [5,6,8,9]. However, it is desirable to explore undiscovered properties by machine learning approaches to further advance prediction accuracy and understand the binding mechanism.

2) Representation of sequences. How to effectively represent sequences of variable lengths as a feature vector using physicochemical properties plays an important role in advancing prediction accuracy. The pseudo-amino acid composition is an efficient representation method of coupling physicochemical properties, which was used to represent a sequence as a 40-dimensional feature vector for discriminating DNA-binding from non-binding proteins [5]. The combined descriptor was proposed using amino acid composition and a series of associated physicochemical properties to form a 132-dimensional feature vector [9]. The conjoint triad descriptor of using a 343-dimensional feature vector was proposed that 20 amino acids were clustered into seven classes according to their dipoles and volumes of side chains [8].

3) Values of amino acids for specific physicochemical properties. The AAindex database [10,11] collected 531 physicochemical and biochemical properties (ignoring 13 properties without available values) with corresponding values of amino acids. How to select an effective feature vector of 20 amino acid values to represent a given property by using experience of designers is not straightforward. Recently, some computational methods of predicting protein functions were successfully developed by mining informative physicochemical properties with their corresponding values from AAindex [12,13].

4) Tradeoffs between prediction accuracy and knowledge acquisition. Besides pursuit of high prediction accuracy, discovering potential properties to further understand the binding mechanism should be also taken into account. Although ensemble classifiers with hybrid feature types and boosting techniques are commonly used to improve prediction performance, it is more desirable to find effective, interpretable physicochemical properties with strong discrimination abilities under using a simple SVM classifier.

This study presents a troublesome problem in using the AAindex database and proposes an effective method to solve. We found that some different physicochemical properties have similar vectors of representing 20 amino acids and some closely related physicochemical properties have dissimilar vectors. For example, several dissimilar vectors in AAindex are available in coupling the hydrophobicity property to represent a sequence and their corresponding performance is significantly different. Similarly, if a different property with a similar vector replaces the known one without significantly degrading prediction performance, it means that the replaced property may be also relative to the binding mechanism from the viewpoint of machine learning. The detailed explanation by using a real quantization example is described below.

**Figure 1:**
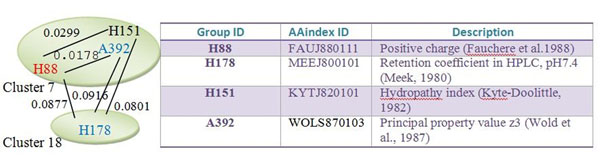
**An illustration example**. The properties H88 and A392 are two different properties but their distance 0.0178 is relatively small. On the other hand, H88 and H178 belonging to the same group Hydrophobicity in AAindex have a large distance 0.0877. H88 and H151 in the same group have a larger distance 0.0299 than that between H88 and A392.

**Figure 2:**
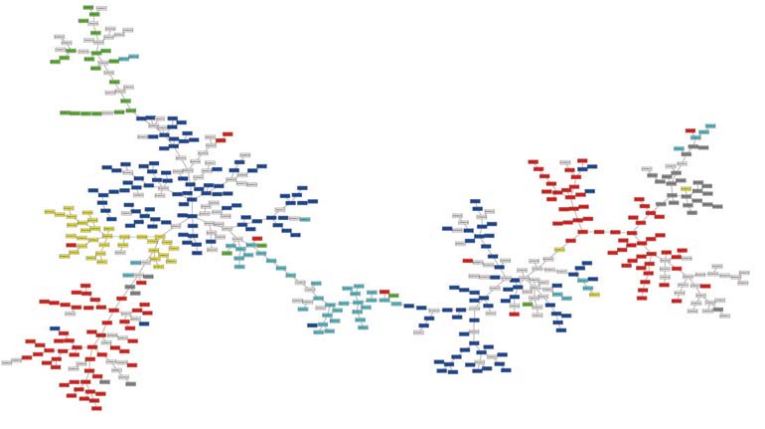
**The minimum spanning tree of the amino acid indices stored in the AAindex1 release 9.0 **[10]. Each rectangle is an amino acid index. Coloured nodes represent the indices classified by Tomii and Kanehisa [11] Red (A): alpha and turn propensities, Yellow (B): beta propensity, Green (C): composition, Blue (H): hydrophobicity, Cyan (P): physicochemical properties, Gray (O): other properties. White: the indices added to the AAindex after the release 3.0 by Tomii and Kanehisa [11].

Figure 1 shows an illustration example. The 402 properties in AAindex were classified into six groups according to their biological meanings classified by Tomii and Kanehisa [11], as shown in Fig. 2 obtained from [10]. According to the numerical indices representing 531 properties of amino acids, we clustered them into 20 clusters by using a fuzzy c-means algorithm based on their Euclidean distances between two indices [14]. The properties H88 and A392 are two different properties belonging to the same cluster 7, but their distance of 0.0178 is relatively small. On the other hand, H88 and H178 belonging to the same group, named Hydrophobicity in AAindex, have a large distance of 0.0877 belonging to clusters 7 and 18, respectively. Although H88 and H151 (used in [5]) are in the same group Hydrophobicity and the same cluster 7, their distance 0.0299 is also larger than that between H88 and A392.

For the aim of designing accurate prediction methods, the major concern is to identify feature vectors with high discrimination abilities for classifying positive and negative samples. This task can be done well for computational methods by using an optimization approach to feature selection from a large set of candidate features. If the feature vectors were identified by predetermined properties based on prior knowledge, the selected vectors of representing amino acids may be not the best. Considering the other aim of discovering potential properties to further look insight the binding mechanism, we propose a systematic, optimization approach (named Auto-IDPCPs) to automatically identify a set of feature vectors and analyze the feature vectors to find properties of affecting the DNA-binding mechanism.

The proposed approach Auto-IDPCPs can identify a small number *m* of feature vectors cooperated with a single SVM classifier, and discover the related hydrophobicity properties with comparable performance, compared with the PSSM feature. Auto-IDPCPs would help designers to investigate informative physicochemical and biochemical properties by considering both prediction accuracy and analysis of binding mechanism simultaneously. Auto-IDPCPs can be also applicable to predict and analyze other protein functions from sequences.

## Methods

**Figure 3:**
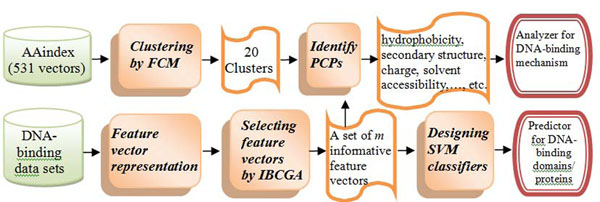
The flowchart of the proposed approach Auto-IDPCPs

The flowchart of the proposed approach Auto-IDPCPs is shown in Fig. 3. The input of the method comprises the AAindex database and three data sets, including DNA-binding domains and sequences, and one independent test data set. The output has two parts: 1) a predictor for DNA-binding domains/proteins with a set of *m* informative feature vectors and the parameter setting of SVM by using an efficient feature selection algorithm IBCGA, and 2) an analyzer with a set of promising physicochemical and biochemical properties in the AAindex database for analyzing the DNA-binding mechanism.

### Data sets

To evaluate effectiveness of the identified physicochemical properties by comparing with the famous PSSM features, we used the benchmark data sets used in the PSSM-400 method [7], as shown in Table 2. The data set DNAset has 146 non-redundant DNA-binding domains (or protein chains) and 250 non-binding domains. No two domains have similarity more than 25%. The data set DNAaset consists of 1153 DNA-binding proteins and 1153 non-binding proteins. No two proteins have similarity more than 25%. An independent data set DNAiset is additionally used, having 92 DNA-binding domains and 100 non-DNA-binding proteins [7].

**Table 2:** The statistic data of the three data sets

Datasets	Sequence	No. of DNA-binding	No. of non-DNA-binding
DNAset	domain	146	250
DNAaset	protein	1153	1153
DNAiset	domain / protein	92	100

### Feature vector representation

All the domains/sequences have a variable length *l*. A sequence forms an *l*-dimensional profile where the value of each amino acid is obtained from the AAindex database for encoding a specific physicochemical property. The *l*-dimensional profiles are transformed into vectors with the same constant length L for utilizing SVM. The transformation can be any known effective representation provided that the L features can effectively classify the *l*-dimensional profiles of positive and negative sequences. The simplest feature is the mean of the profile that L=1 [12,13]. Therefore, the sequences with *m* properties are represented as an *m*-dimensional feature vectors. Finally, all values of the feature vectors are normalized into [-1, 1] to apply SVM.

### Feature selection by IBCGA

Selecting a minimal number of informative features while maximizing prediction accuracy is a bi-objective 0/1 combinatorial optimization problem. An efficient inheritable bi-objective combinatorial genetic algorithm IBCGA [15] bases on an intelligent genetic algorithm IGA [16] is utilized to solve this optimization problem. IGA based on orthogonal experimental design uses a divide-and-conquer strategy and a systematic reasoning method instead of the conventional generate-and-go method to efficiently solve the combinatorial optimization problem *C*(*n*, *m*) having a huge search space of size *n*!/(*m*!(*n-m*)!)), where *n*=531 in this study. IBCGA can efficiently search the space of C(*n*, *r*±1) by inheriting a good solution in the space of C(*n*, *r*) [15]. Therefore, IBCGA can economically obtain a complete set of high-quality solutions in a single run.

The chromosome encoding scheme of IGA consists of *n* binary IGA-genes *g_i_* for selecting informative features (physicochemical properties) and two 4-bit IGA-genes for encoding *γ* and *C* of SVM parameters, where *γ*∈{2^-7^, 2^-6^…, 2^8^} and *C*∈{2^-7^, 2^-6^…, 2^8^} [12]. The *i*^th^ feature is used in the SVM classifier if *g_i_*=1; otherwise, the *i*^th^ feature is excluded (*g_i_*=0). The performance of selected properties associated with the parameter values of SVM is measured by five-fold cross-validation (5-CV) for comparing with the method PSSM-400 [7]. IBCGA with the fitness function *f*(X) of 5-CV prediction accuracy can simultaneously obtain a set of solutions, X*_r_*, where *r*=*r*_start_, *r*_start_+1, …, *r*_end_ in a single run. In this study, the parameter settings *r*_start_ =10, *r*_end_ =30, *N*_pop_ =50, *p_c_* =0.8, and *p_m_* =0.05. The output contains a set of *m* selected properties from AAindex and an SVM classifier with associated parameter settings *γ* and *C*. The customized IBCGA algorithm used in Auto-IDPCPs is given below.

Step 1:	(Initiation) Randomly generate an initial population of *N*_pop_ individuals. All the *n* binary genes have *r* 1’s and *n*-*r* 0’s where *r* = *r*_start_.

Step 2:	(Evaluation) Evaluate the fitness values of all individuals using *f*(X).

Step 3:	(Selection) Use the traditional tournament selection that selects the winner from two randomly selected individuals to form a mating pool.

Step 4:	(Crossover) Select *p_c_*·*N*_pop_ parents from the mating pool to perform orthogonal array crossover on the selected pairs of parents where *p_c_* is the crossover probability.

Step 5:	(Mutation) Apply the mutation operator to the randomly selected *p_m_*·*N*_pop_ individuals in the new population where *p_m_* is the mutation probability. To prevent the best fitness value from deteriorating, mutation is not applied to the best individual.

Step 6:	(Termination test) If the stopping condition for obtaining the solutions X*_r_* is satisfied, output the best individual as X*_r_*. Otherwise, go to Step 2.

Step 7:	(Inheritance) If r <*r*_end_, randomly change one bit in the binary genes for each individual from 0 to 1; increase the number *r* by one, and go to Step 2.

Step 8:	 (System uncertainty) Perform Steps 1-7 for *R* (=30 in this study) independent runs and obtain the best one of *R* solutions. The best solution can be determined by considering the most accurate one *S*_a_ with the highest prediction accuracy or the robust one *S*_r_ with the highest score, where the scores *S_t_*, *t*=1, …, *R* are derived using the following procedure Appscore.

The procedure Appscore is described as follows:

Step 1: Calculate the appearance frequency f(*p_i_*) of each feature *p_i_* from all features in the *R* sets, where *i*=1, …, *m_t_*. There are *m_Â¬t_* features in the *t*-th set.

Step 2: Calculate score *S_t_*_,_*t*=1, …, *R* for each of *R* solutions:(1)

### Clustering properties by fuzzy c-means

The physicochemical properties can be classified into six groups according to their biological meanings [11]. From the viewpoint of machine learning, two properties are similar if the distance between their feature vectors is small. To identify informative physicochemical properties and obtain effective feature vectors with strong discriminative abilities, we cluster the 531 vectors of physicochemical properties into 20 clusters using a fuzzy c-means (FCM) method [14].

A feature vector of amino acids is a set of 20 numerical values representing a physicochemical property of amino acids. To apply the FCM method, all data were normalized in such a way that every physicochemical property had an averaged profile value of zero and a standard deviation equal to 1. The FCM method has an objective functional of the form [14]:(2)

where *n=*531 is the number of data vectors, *K* is the number of clusters to be found, *u_ij_*∈[0, 1] is the membership degree of *j^th^* data vector *x_j_* in the *i^th^* cluster, the *i^th^* cluster represented by the cluster prototype *v_i_*, *s*∈[1,∞) is a weighting exponent called the fuzzifier and *d*(*v_i_*, *x_j_*) is the distance of *x_j_* from the cluster prototype *v_i_*. Dembélé and Kastner [17] suggested the parameters setting *s*=1.12 and *K=*20 clusters, adopted in this study.

### Identifying physicochemical properties

It is not easy to discover related physicochemical properties for analyzing DNA-binding mechanism by computational methods using relatively small-size data sets. Therefore, we present a hybrid method by combining evidences from the viewpoints of both machine learning and biological meanings. Auto-IDPCPs identifies *m* properties belong to *c* out of 20 clusters. We examine each property P1 of the 531 properties and each property P2 of the identified *m* properties. If they satisfy the following two criteria, P1 is a promising property to be further investigated: 1) the distance between the feature vectors of P1 and P2 is small, and 2) P2 is replaced with P1 one at a time and the prediction accuracy is not significantly decreased.

**Figure 4:**
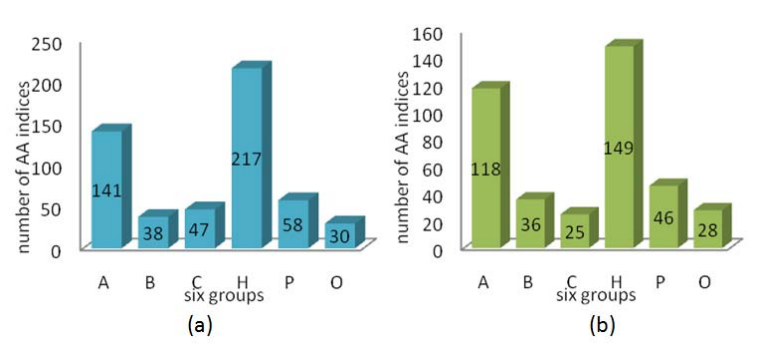
The statistical results of property distribution in the six groups (a) 531 amino acid indices (b) 402 amino acid indices

Only 402 of 531 properties were classified into six groups [11], (A): Alpha and turn propensities, (B): Beta propensity, (C): Composition, (H): Hydrophobicity, (P): Physicochemical properties, and (O): Other properties. We classified the other 129 properties into the six groups according to their distances of vectors using a nearest-neighbour rule. The 531 featured IDs and their corresponding AAindex IDs are listed in Table S1 (see Additional File 1). The 20 clusters and their corresponding physicochemical and biochemical properties belonging to six groups in the AAindex database are given in Table 3. The statistical result of the 531 physicochemical properties distributed upon the 20 clusters and six groups is given in Table 4. The large clusters 7, 9 and 10 have properties belonging to six groups. The valuable information in Tables 3 and 4 integrating classification results of both biological meanings and similarity measure is very helpful in identifying informative properties. The statistical results of property distribution in the six groups for 531 and 402 amino acid indices are given in Fig. 4. The largest group is Hydrophobicity having 149 indices and the second largest group is Alpha and turn propensities having 118 indices (Fig. 4b). The sizes of the other four groups are relatively small.

**Table 3:** The 20 clusters and their corresponding physicochemical and biochemical properties in the AAindex database

Cluster	No.	The label of 531 physicochemical and biochemical properties
C_1_	2	P: 118 O: 156

C_2_	2	P: 504 505

C_3_	6	H: 10 11 446 447 448 449

C_4_	3	P: 9 112 150

C_5_	4	C: 116 H: 34 127 P: 117

C_6_	6	A: 313 H: 129 145 364 P: 177 O: 312

C_7_	147	A: 19 25 49 50 52 74 166 258 259 260 261 262 263 264 265 266 267 268 269 270 274 286 287 288 289 290 291 292293 294 295 296 341 346 347 348 350 351 359 362 376 392 424 454 455487 524 B: 20 196 221 275 276 277 278 279 C: 30 134 H: 8 14 35 36 57 68 71 85 88 89 113 115 131 146 149 151 152 153 198 210 213 215 220 239 240 249 271 272273 283 284 285 314 317 318 320 340 356 360 365 377 378 379 380 381 386 387 389 390 435 438 439 476 484 489 490 503 507 509 510 511 512 513 514 516 518 520 525 526 527 528 529 531 P: 22 159 214 216 217 280 281 282 316 361 397 485 486 488 O: 51 250 385

C_8_	3	P: 65 135 517

C_9_	132	A: 5 7 24 37 40 44 47 48 53 62 93 104 105 107 121 122 124 162 165 176 188 227 228 229 230 235 236 237 238 255 303 309 334 335 337 338 345 367 369 375 413 417 418 420 428 429 430 432 433 436 498 B: 106 C: 31 144 H: 6 41 86 87 92 94 95 110 114 125 142 143 182 183 297 298 299 300 302 325 326 327 354 355 357 371 373 384 403 404 405 406 407 408 421 422 425 427 431 453 482 491 492 493 494 496 497 499 506 530 P: 15 28 59 219 383 391 O: 21 27 43 79 123 126 173 174 175 251 301 305 308 322 323 324 336 370 372 374 409 419

C_10_	123	A: 38 42 60 91 97 98 99 100 119 138 140 160 163 171 186 223 224 231 253 256 307 311 328 330 331 333 339 342 349 363 366 410 411 412 414 415 416 426 B: 39 45 46 61 101 102 103 120 139 141 161 164 167 168 169 172 187 218 225 226 232 234 254 257 310 343 344 368 445 495 C: 190 304 H: 2 3 4 23 54 55 56 58 66 67 69 70 76 77 108 130 132 133 192 194 201 211 212233 243 315 321 329 332 352 358 398 477 478 480 481 508 515 519 521 522 523 P: 26 29 78 82 96 154 157 473 479 O: 306 437

C_11_	6	P: 32 72 109 353 474 475

C_12_	2	H: 128 483

C_13_	1	C: 137

C_14_	15	H: 170 241 244 245 246 393 395 396 400 402 423 444 P: 399 502 O: 84

C_15_	1	H: 73

C_16_	43	A: 18 C: 64 136 189 191 193 195 197 199 200 202 203 204 205 206 207 208 209 440 441 442 443 456 457 458 459 460 461 462 463 464 465 466 467 468 469 470 471 472 H: 147 148 222 252

C_17_	3	H: 450 451 452

C_18_	28	A: 16 90 H: 12 13 111 178 179 180 181 185 242 247 248 382 388 394 401 500 501 P: 1 17 63 80 81 83 158 434 O: 75

C_19_	2	H: 184 O: 155

C_20_	2	P: 33 319

A: Alpha and turn propensities. B: Beta propensity. C: Composition. H: Hydrophobicity. P: Physicochemical properties. O: Other properties.

**Table 4:** The statistical result of the 531 physicochemical properties distributed upon the 20 clusters and six groups

Cluster	A	B	C	H	P	O	TOTAL
C_1_					1	1	2
C_2_					2		2
C_3_				6			6
C_4_					3		3
C_5_			1	2	1		4
C_6_	1			3	1	1	6
C_7_	47	7	2	74	14	3	147
C_8_					3		3
C_9_	51	1	3	50	6	21	132
C_10_	38	30	2	42	9	2	123
C_11_					6		6
C_12_				2			2
C_13_			1				1
C_14_				12	2	1	15
C_15_				1			1
C_16_	1		38	4			43
C_17_				3			3
C_18_	3			17	8		28
C_19_				1		1	2
C_20_					2		2
TOTAL	141	38	47	217	58	30	531
RATE	0.266	0.072	0.089	0.409	0.109	0.056	

A: Alpha and turn propensities. B: Beta propensity. C: Composition. H: Hydrophobicity. P: Physicochemical properties. O: Other properties.

## Results

### Identified properties by IBCGA

**Figure 5:**
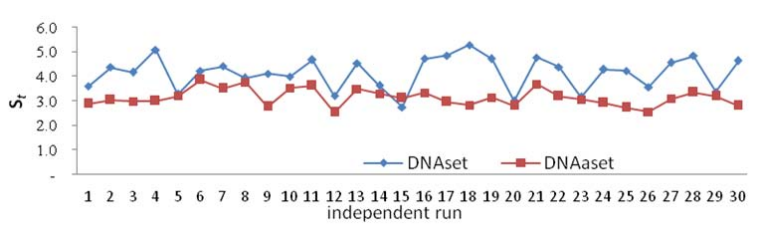
The statistical result of S_t_ in selecting property sets from *R* =30 independent runs on DNAset and DNAaset.

**Figure 6:**
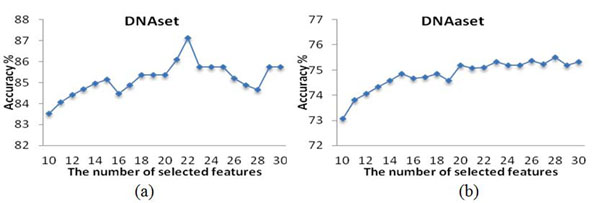
Prediction accuracies for various numbers of selected properties (a) DNAset and (b) DNAaset.

The statistical result of S_t_ in selecting property sets from *R* =30 independent runs on DNAset and DNAaset is given in Fig. 5. The 18^th^ and 6^th^ runs having the highest scores for DNAset and DNAaset respectively are selected, and their prediction accuracies for various numbers of selected features are given in Fig. 6. The robust solutions S_18_ and S_6_ having *m*=22 and 28 features with accuracies of 87.12% and 75.50% for DNAset and DNAaset, respectively, are given in Tables 5 and 6, where the AAindex identity numbers and their property descriptions are provided.

**Table 5:** The robust solution S_18_ with a set of *m*=22 features for DNAset

Feature ID	AAindex ID	Description
**53**	CHOP780216	Normalized frequency of the 2nd and 3rd residues in turn (Chou-Fasman, 1978b)
**56**	CIDH920103	Normalized hydrophobicity scales for alpha+beta-proteins (Cid et al., 1992)
**64**	DAYM780101	Amino acid composition (Dayhoff et al., 1978a)
**86**	FAUJ880109	Number of hydrogen bond donors (Fauchere et al., 1988)
**91**	FINA770101	Helix-coil equilibrium constant (Finkelstein-Ptitsyn, 1977)
**188**	NAGK730103	Normalized frequency of coil (Nagano, 1973)
**202**	NAKH920101	AA composition of CYT of single-spanning proteins (Nakashima-Nishikawa, 1992)
**227**	PALJ810105	Normalized frequency of turn from LG (Palau et al., 1981)
**228**	PALJ810106	Normalized frequency of turn from CF (Palau et al., 1981)
**255**	PRAM900104	Relative frequency in reverse-turn (Prabhakaran, 1990)
**262**	QIAN880105	Weights for alpha-helix at the window position of -2 (Qian-Sejnowski, 1988)
**274**	QIAN880117	Weights for beta-sheet at the window position of -3 (Qian-Sejnowski, 1988)
**286**	QIAN880129	Weights for coil at the window position of -4 (Qian-Sejnowski, 1988)
**363**	SUEM840101	Zimm-Bragg parameter s at 20 C (Sueki et al., 1984)
**383**	WEBA780101	RF value in high salt chromatography (Weber-Lacey, 1978)
**388**	WOEC730101	Polar requirement (Woese, 1973)
**412**	AURR980110	Normalized positional residue frequency at helix termini N5 (Aurora-Rose, 1998)
**430**	MUNV940102	Free energy in alpha-helical region (Munoz-Serrano, 1994)
**434**	WIMW960101	Free energies of transfer of AcWl-X-LL peptides from bilayer interface to water (Wimley-White, 1996)
**443**	KUMS000104	Distribution of amino acid residues in the alpha-helices in mesophilic proteins (Kumar et al., 2000)
**486**	BASU050102	Interactivity scale obtained by maximizing the mean of correlation coefficient over single-domain globular proteins (Bastolla et al., 2005)
**513**	JACR890101	Weights from the IFH scale (Jacobs-White, 1989)

**Table 6:** The robust solution S_6_ with a set of *m*=28 features for DNAaset

Feature ID	AAindex ID	Description
**39**	CHOP780202	Normalized frequency of beta-sheet (Chou-Fasman, 1978b)
**56**	CIDH920103	Normalized hydrophobicity scales for alpha+beta-proteins (Cid et al., 1992)
**58**	CIDH920105	Normalized average hydrophobicity scales (Cid et al., 1992)
**86**	FAUJ880109	Number of hydrogen bond donors (Fauchere et al., 1988)
**88**	FAUJ880111	Positive charge (Fauchere et al., 1988)
**95**	FINA910104	Helix termination parameter at posision j+1 (Finkelstein et al., 1991)
**100**	GEIM800104	Alpha-helix indices for alpha/beta-proteins (Geisow-Roberts, 1980)
**102**	GEIM800106	Beta-strand indices for beta-proteins (Geisow-Roberts, 1980)
**139**	KANM800102	Average relative probability of beta-sheet (Kanehisa-Tsong, 1980)
**146**	KLEP840101	Net charge (Klein et al., 1984)
**147**	KRIW710101	Side chain interaction parameter (Krigbaum-Rubin, 1971)
**167**	LIFS790101	Conformational preference for all beta-strands (Lifson-Sander, 1979)
**178**	MEEJ800101	Retention coefficient in HPLC, pH7.4 (Meek, 1980)
**214**	OOBM770102	Short and medium range non-bonded energy per atom (Oobatake-Ooi, 1977)
**229**	PALJ810107	Normalized frequency of alpha-helix in all-alpha class (Palau et al., 1981)
**280**	QIAN880123	Weights for beta-sheet at the window position of 3 (Qian-Sejnowski, 1988)
**299**	RACS770103	Side chain orientational preference (Rackovsky-Scheraga, 1977)
**321**	RADA880108	Mean polarity (Radzicka-Wolfenden, 1988)
**356**	ROSM880102	Side chain hydropathy, corrected for solvation (Roseman, 1988)
**365**	SWER830101	Optimal matching hydrophobicity (Sweet-Eisenberg, 1983)
**399**	ZIMJ680102	Bulkiness (Zimmerman et al., 1968)
**401**	ZIMJ680104	Isoelectric point (Zimmerman et al., 1968)
**422**	AURR980120	Normalized positional residue frequency at helix termini C4' (Aurora-Rose, 1998)
**431**	MUNV940103	Free energy in beta-strand conformation (Munoz-Serrano, 1994)
**449**	NADH010104	Hydropathy scale based on self-information values in the two-state model (20% accessibility) (Naderi-Manesh et al., 2001)
**451**	NADH010106	Hydropathy scale based on self-information values in the two-state model (36% accessibility) (Naderi-Manesh et al., 2001)
**512**	GUYH850105	Apparent partition energies calculated from Chothia index (Guy, 1985)
**528**	MIYS990104	Optimized relative partition energies - method C (Miyazawa-Jernigan, 1999)

**Figure 7:**
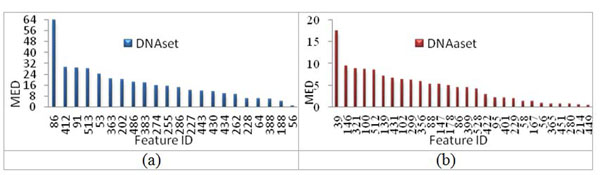
The *m* properties ranked by using main effect difference (MED) (a) m=22 for DNAset and (b) m=28 for DNAaset.

An efficient way to study effects of several factors simultaneously is to utilize orthogonal experimental design used in IGA [16]. The main effect difference (MED) of orthogonal experimental design can estimate effects of individual features according to the value of MED [12,16]. The most effective property has the largest value of MED. The *m* properties ranked by using MED are shown in Fig. 7. The properties of rank 1 are identity numbers H86 (FAUJ880109, Number of hydrogen bond donors) and B39 (CHOP780202, Normalized frequency of beta-sheet) for DNAset and DNAaset, belonging to the groups Hydrophobicity and Beta propensity, respectively.

### Prediction performance evaluation

To evaluate the effectiveness of the identified *m* informative feature vectors (PCPs), we implemented the predictor using the same single classifier SVM with the feature types, amino acid composition (AAC) and PSSM [7]. Additionally, the selected PCPs combined with AAC and PSSM were also evaluated, as shown in Table 7. AAC is a 20-dimensional vector of amino acid composition. PSSM is the feature representation of 400 features. PCPs +AAC and PCPs +PSSM are two hybrid feature types.

Considering the DNA-binding domain data set DNAset, the set of *m*=22 informative properties (PCPs) identified by Auto-IDPCPs performs best where the robust solution *S_r_* with accuracy of 87.12% is used. The four compared methods AAC, PSSM, PCPs+AAC, and PCPs+PSSM have accuracies of 80.30%, 86.62%, 81.82% and 86.62%, respectively. For the DNA-binding protein data set DNAaest, the method using PCPs with *m*=28 informative properties (75.50%) is slightly better than the PSSM-400 (74.22%). However, PCPs+PSSM can improve both PCPs and PSSM to the accuracy of 80.27%. When the method using the trained SVM with PCPs was evaluated by the independent test data set DNAiset, the method using selected PCPs has the accuracy of 80.73% (=155/192), slightly worse than the method PSSM-400 with the accuracy of 82.81% (=159/192) [7].

**Table 7:** The overall accuracies (%) of 5-CV for three feature types and two hybrid feature types with SVM

Dataset		Sen.	Spe.	MCC	PCPs	AAC	PSSM*	PCPs +AAC	PCPs +PSSM
DNAset	*S_a_*	88.89	91.20	0.76	88.89	80.30	86.62	81.57	83.59
	
	*S_r_*	82.19	90.00	0.53	87.12			81.82	86.62

DNAaest	*S_a_*	82.74	70.08	0.72	76.41	72.46	74.22	74.20	79.88
	
	*S_r_*	81.96	69.04	0.51	75.50			73.59	80.27

*S_a_*: accurate solution, *S_r_*: robust solution, Sen.: sensitivity, Spe.: specificity, MCC: Matthew’s correlation coefficient, PCPs: the *m* informative properties, PSSM*: obtained from [7].

A small, high-performance features set of size c that one property is selected from each of the identified c clusters using the optimization algorithm IGA is given in Table 8, where c=5 and 8 for DNAset and DNAaset, respectively. The accuracies of the robust solutions using the c features with SVM are 83.59% and 73.24% for DNAset and DNAaset, respectively. The properties and their descriptions are given in Tables 9 and 10.

The experimental results reveal that the identified small set of *m* physicochemical properties with a simple sequence representation and a single SVM classifier is promising, compared with the PSSM feature type. However, the identified physicochemical properties are interpretable for further understanding the DNA-binding mechanism.

**Table 8:** A small, high-performance features set of size c from c clusters. The feature number c=5 and 8 for DNAset and DNAaset, respectively.

DNAset	ACC 83.59%	Cluster	C*_7_*	C*_9_*	C*_10_*	C*_16_*	C*_18_*			
		Feature ID	H88	H86	H67	H209	H178			
DNAaset	ACC 73.24%	Cluster	C*_7_*	C*_9_*	C*_10_*	C*_16_*	C*_18_*	C*_3_*	C*_14_*	C*_17_*
		Feature ID	P159	H87	A99	C197	P63	H11	H396	H451

**Table 9:** The m=5 features selected from the five clusters identified from DNAset that one best feature is selected from one cluster by IGA.

Feature ID	AAindex ID	Description
H88	FAUJ880111	Positive charge (Fauchere et al., 1988)
H86	FAUJ880109	Number of hydrogen bond donors (Fauchere et al., 1988)
H67	DESM900102	Average membrane preference: AMP07 (Degli Esposti et al., 1990)
C209	NAKH920108	AA composition of MEM of multi-spanning proteins (Nakashima-Nishikawa, 1992)
H178	MEEJ800101	Retention coefficient in HPLC, pH7.4 (Meek, 1980)

**Table 10:** The m=8 features selected from the eight clusters identified from DNAaset that one best feature is selected from one cluster by IGA.

Feature ID	AAindex ID	Description
P159	LEVM760107	van der Waals parameter epsilon (Levitt, 1976)
H87	FAUJ880110	Number of full nonbonding orbitals (Fauchere et al., 1988)
A99	GEIM800103	Alpha-helix indices for beta-proteins (Geisow-Roberts, 1980)
C197	NAKH900109	AA composition of membrane proteins (Nakashima et al., 1990)
P63	DAWD720101	Size (Dawson, 1972)
H11	BIOV880102	Information value for accessibility; average fraction 23% (Biou et al., 1988)
H396	YUTK870104	Activation Gibbs energy of unfolding, pH9.0 (Yutani et al., 1987)
H451	NADH010106	Hydropathy scale based on self-information values in the two-state model (36% accessibility) (Naderi-Manesh et al., 2001)

### Analyzing physicochemical properties for binding mechanism

**Figure 8:**
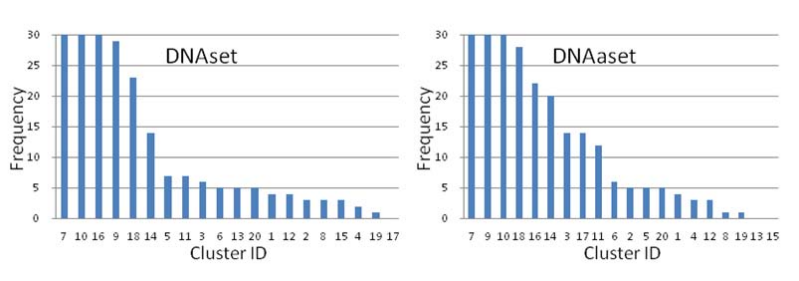
The appearance frequency of each identified cluster in the 30 runs. The clusters 7, 9, 10, 16 and 18 are more informative.

The selected features of the conducted 30 runs are very different in terms of feature ID from 531 properties. The appearance frequency of each identified cluster in the 30 runs is shown in Fig. 8. From the statistic result, the clusters 7, 9, 10, 16 and 18 with very high selection frequencies are more informative for predicting DNA-binding domains and proteins. The selected clusters of the 30 runs are very similar in terms of cluster ID from 20 clusters. The *m*=22 properties (Table 5) belong to five clusters which are the same with the five clusters 7, 9, 10, 16 and 18. For predicting DNA-binding proteins, the *m*=28 properties (Table 6) belong to eight clusters, which are the same five clusters and additional three clusters 3, 14 and 17. The results reveal that the identified features of the robust solution S_r_ belong to informative clusters. Therefore, the informative clusters can provide informative properties to be further investigated if these properties were not selected by the feature selection method.

**Figure 9:**
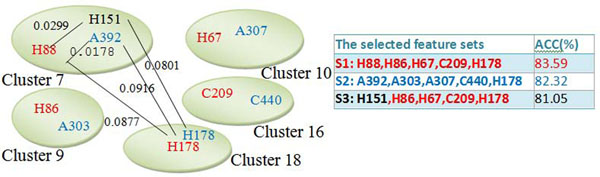
An illustration example for exploring promising properties. H151 can be inferred from feature sets S1 and S2.

An illustration example for exploring promising properties is given in Fig. 9. The both feature sets S1 (H88, H86, H67, C209, H178) and S2 (A392, A303, A307, C440, H178) are selected for predicting DNA-binding domains in DNAset that one property is selected from one of five clusters 7, 9, 10, 16 and 18. The identified properties H88 and A392 belong to the Hydrophobicity, and Alpha and turn propensities groups respectively, but they belong to the same cluster 7 with a relatively small distance 0.0178. The prediction accuracy of S3 by replacing H88 with H151 is 81.05 %. On the other hand, H151 belonging to the cluster 7 and Hydrophobicity group used in [5] can be inferred from feature sets S1 and S2. After analyzing all candidate properties, we identify some properties in the five identified clusters for analyzing DNA-binding domains, shown in Table 11. Some typical properties discovered are hydrophobicity, secondary structure, charge, solvent accessibility, polarity, flexibility, normalized Van Der Waals volume, pK (pK-C, pK-N, pK-COOH and pK-a(RCOOH)), etc.

**Table 11:** Some typical properties in the five identified clusters for analyzing DNA-binding domains

C_id _AAindex ID PCP		C_id _AAindex ID PCP		
7	BHAR880101 Flexibility	10	FASG760105	pK-C
7	BURA740101 Secondary structure	10	JOND750102	pk- (-COOH)
7	CHOC760103 Solvent accessibility	10	RADA880108	Polarity
7	HOPT810101 Hydrophobicity	16	PRAM900101	Hydrophobicity
7	FAUJ880111 Charge	16	FUKS010104	Solvent accessibility
9	KARP850101 Flexibility	16	KUMS000103	Secondary structure
9	PALJ810115 Secondary structure	18	PONP800107	Solvent accessibility
9	ROSM880101 Hydrophobicity	18	GRAR740102	Polarity
9	KUHL950101 Solvent accessibility	18	FASG760104	pK-N
10	ZIMJ680101 Hydrophobicity	18	FAUJ880113	pK-a(RCOOH)
10	EISD860101 Solvent accessibility	18	FAUJ880103	Normalized van der
10	GEIM800101 Secondary structure			Waals volume

C_id_: Cluster ID. PCP: physicochemical and biochemical property

Most of identified properties were used in previous works [5,6,8,9], but a few properties such as the flexibility property H8: BHAR880101 in cluster 7 “Average flexibility indices (Bhaskaran-Ponnuswamy, 1988)” are not utilized yet in existing method of predicting DNA-binding domains. The correlation between protein flexibility and protein function suggests a link between DNA-binding activity and the conformational freedom of the DNA-binding domain [18].

Although most identified properties were mentioned in literature, it is not easy to know which amino acid index is the best feature in designing accurate prediction method. For example, the two feature vectors of properties H8: BHAR880101 and A:124 KARP850101 “Flexibility parameter for no rigid neighbours (Karplus-Schulz, 1985)” belonging to different clusters 7 and 9 are dissimilar. From Table 11, it can be found that the four clusters 7, 9, 10 and 16 has properties, H115: HOPT810101 (Hydrophilicity value (Hopp-Woods, 1981)), H355: ROSM880101 (Side chain hydropathy, uncorrected for solvation (Roseman, 1988)), H398: ZIMJ680101 (Hydrophobicity (Zimmerman et al., 1968)), and H252: PRAM900101 (Hydrophobicity (Prabhakaran, 1990)), respectively, belonging to the Hydrophobicity group. The scenario reveals that more than one feature vectors may be needed in utilizing one physicochemical property to advance prediction accuracy.

## Discussion

To avoid from overfitting the small-scale data sets in identifying physicochemical properties using an optimization approach, this study proposes a hybrid computational method of combining evidences by considering robust features and biological meanings from literature. These discovered properties in predicting and analyzing the DNA-binding mechanism can be further investigated.

In this study, the proposed approach Auto-IDPCPs aims to identify an informative feature set of physicochemical and biochemical properties, rather than proposing an accurate method for predicting DNA-binding domains/proteins. Some techniques can be used to improve the prediction accuracy such as using ensemble classifiers with hybrid feature types and boosting techniques.

### Conclusions

This study has proposed a systematic approach Auto-IDPCPs to automatically identify an informative set of physicochemical and biochemical properties in the AAindex database to design SVM-based classifiers for predicting and analyzing DNA-binding domains/proteins. Since the AAindex database contains 531 physicochemical and biochemical properties of 20 naturally occurring amino acids that are the building-blocks of proteins, the proposed approach Auto-IDPCPs using an optimization method of feature selection can be useful and efficient for selecting informative physicochemical and biochemical properties, which is helpful in designing prediction methods for protein functions from sequences.

## Authors' contributions

HLH designed the system, implemented programs, carried out the analysis, and participated in manuscript preparation. ICL provided biological knowledge and carried out the analysis. YFL, CTT, KTH, WLH and SJH implemented programs and participated in the experimental design. SYH supervised the whole project and participated in manuscript preparation. All authors have read and approved the final manuscript.

## Competing interests

The authors declare that they have no competing interests.

## Supplementary Material

Additional file 1 Table S1 - The 531 feature IDs used in this study and their corresponding AAindex IDs. Click here for file
