# Supplementary material for: Predicting and analyzing DNA-binding domains using a systematic approach to identifying a set of informative physicochemical and biochemical properties
Source: BMC Bioinformatics. 2011 Feb 15;12(Suppl 1):S47. doi: 10.1186/1471-2105-12-S1-S47 (PMC3044304; doi:10.1186/1471-2105-12-S1-S47)
Supplement: Additional file 1 — Table S1 - The 531 feature IDs used in this study and their corresponding AAindex IDs. [file 1471-2105-12-S1-S47-S1.doc]

**Supplementary Table**

## Table S1 - The 531 feature IDs used in this study and their corresponding AAindex IDs.

| Feature  ID  1 ANDN920101  2 ARGP820101  3 ARGP820102  4 ARGP820103  5 BEGF750101  6 BEGF750102  7 BEGF750103  8 BHAR880101  9 BIGC670101  10 BIOV880101  11 BIOV880102  12 BROC820101  13 BROC820102  14 BULH740101  15 BULH740102  16 BUNA790101  17 BUNA790102  18 BUNA790103  19 BURA740101  20 BURA740102  21 CHAM810101  22 CHAM820101  23 CHAM820102  24 CHAM830101  25 CHAM830102  26 CHAM830103  27 CHAM830104  28 CHAM830105  29 CHAM830106  30 CHAM830107  31 CHAM830108  32 CHOC750101  33 CHOC760101  34 CHOC760102  35 CHOC760103  36 CHOC760104  37 CHOP780101  38 CHOP780201  39 CHOP780202  40 CHOP780203  41 CHOP780204  42 CHOP780205  43 CHOP780206  44 CHOP780207  45 CHOP780208  46 CHOP780209  47 CHOP780210  48 CHOP780211  49 CHOP780212  50 CHOP780213  51 CHOP780214  52 CHOP780215  53 CHOP780216  54 CIDH920101  55 CIDH920102  56 CIDH920103  57 CIDH920104  58 CIDH920105  59 COHE430101  60 CRAJ730101  61 CRAJ730102  62 CRAJ730103  63 DAWD720101  64 DAYM780101  65 DAYM780201  66 DESM900101  67 DESM900102  68 EISD840101  69 EISD860101  70 EISD860102  71 EISD860103  72 FASG760101  73 FASG760102  74 FASG760103  75 FASG760104  76 FASG760105  77 FAUJ830101  78 FAUJ880101  79 FAUJ880102  80 FAUJ880103  81 FAUJ880104  82 FAUJ880105  83 FAUJ880106  84 FAUJ880107  85 FAUJ880108  86 FAUJ880109  87 FAUJ880110  88 FAUJ880111  89 FAUJ880112  90 FAUJ880113  91 FINA770101  92 FINA910101  93 FINA910102  94 FINA910103  95 FINA910104  96 GARJ730101  97 GEIM800101  98 GEIM800102  99 GEIM800103  100 GEIM800104  101 GEIM800105  102 GEIM800106  103 GEIM800107  104 GEIM800108  105 GEIM800109  106 GEIM800110  107 GEIM800111  108 GOLD730101  109 GOLD730102  110 GRAR740101  111 GRAR740102  112 GRAR740103  113 GUH850101  114 HOPA770101  115 HOPT810101  116 HUTJ700101  117 HUTJ700102  118 HUTJ700103  119 ISOY800101  120 ISOY800102  121 ISOY800103  122 ISOY800104  123 ISOY800105  124 ISOY800106  125 ISOY800107  126 ISOY800108  127 JANJ780101  128 JANJ780102  129 JANJ780103  130 JANJ790101  131 JANJ790102  132 JOND750101  133 JOND750102  134 JOND920101  135 JOND920102  136 JUKT750101  137 JUNJ780101  138 KANM800101  139 KANM800102  140 KANM800103  141 KANM800104  142 KARP850101  143 KARP850102  144 KARP850103  145 KHAG800101  146 KLEP840101  147 KRIW710101  148 KRIW790101  149 KRIW790102  150 KRIW790103  151 KYTJ820101  152 LAWE840101  153 LEVM760101  154 LEVM760102  155 LEVM760103  156 LEVM760104  157 LEVM760105  158 LEVM760106  159 LEVM760107  160 LEVM780101  161 LEVM780102  162 LEVM780103  163 LEVM780104  164 LEVM780105  165 LEVM780106  166 LEWP710101  167 LIFS790101  168 LIFS790102  169 LIFS790103  170 MANP780101  171 MAXF760101  172 MAXF760102  173 MAXF760103  174 MAXF760104  175 MAXF760105  176 MAXF760106  177 MCMT640101  178 MEEJ800101  179 MEEJ800102  180 MEEJ810101  181 MEEJ810102  182 MEIH800101  183 MEIH800102  184 MEIH800103  185 MIYS850101  186 NAGK730101  187 NAGK730102  188 NAGK730103  189 NAKH900101  190 NAKH900102  191 NAKH900103  192 NAKH900104  193 NAKH900105  194 NAKH900106  195 NAKH900107  196 NAKH900108  197 NAKH900109  198 NAKH900110  199 NAKH900111  200 NAKH900112  201 NAKH900113  202 NAKH920101  203 NAKH920102  204 NAKH920103  205 NAKH920104  206 NAKH920105  207 NAKH920106  208 NAKH920107  209 NAKH920108  210 NISK800101  211 NISK860101  212 NOZY710101  213 OOBM770101  214 OOBM770102  215 OOBM770103  216 OOBM770104  217 OOBM770105  218 OOBM850101  219 OOBM850102  220 OOBM850103  221 OOBM850104  222 OOBM850105  223 PALJ810101  224 PALJ810102  225 PALJ810103  226 PALJ810104  227 PALJ810105  228 PALJ810106  229 PALJ810107  230 PALJ810108  231 PALJ810109  232 PALJ810110  233 PALJ810111  234 PALJ810112  235 PALJ810113  236 PALJ810114  237 PALJ810115  238 PALJ810116  239 PARJ860101  240 PLIV810101  241 PONP800101  242 PONP800102  243 PONP800103  244 PONP800104  245 PONP800105  246 PONP800106  247 PONP800107  248 PONP800108  249 PRAM820101  250 PRAM820102  251 PRAM820103  252 PRAM900101  253 PRAM900102  254 PRAM900103  255 PRAM900104  256 PTIO830101  257 PTIO830102  258 QIAN880101  259 QIAN880102  260 QIAN880103  261 QIAN880104  262 QIAN880105  263 QIAN880106  264 QIAN880107  265 QIAN880108  266 QIAN880109  267 QIAN880110  268 QIAN880111  202 NAKH920101  203 NAKH920102  204 NAKH920103  205 NAKH920104  206 NAKH920105  207 NAKH920106  208 NAKH920107  209 NAKH920108  210 NISK800101  211 NISK860101  212 NOZY710101  213 OOBM770101  214 OOBM770102  215 OOBM770103  216 OOBM770104  217 OOBM770105  218 OOBM850101  219 OOBM850102  220 OOBM850103  221 OOBM850104  222 OOBM850105  223 PALJ810101  224 PALJ810102  225 PALJ810103  226 PALJ810104  227 PALJ810105  228 PALJ810106  229 PALJ810107  230 PALJ810108  231 PALJ810109  232 PALJ810110  233 PALJ810111  234 PALJ810112  235 PALJ810113  236 PALJ810114  237 PALJ810115  238 PALJ810116  239 PARJ860101  240 PLIV810101  241 PONP800101  242 PONP800102  243 PONP800103  244 PONP800104  245 PONP800105  246 PONP800106  247 PONP800107  248 PONP800108  249 PRAM820101  250 PRAM820102  251 PRAM820103  252 PRAM900101  253 PRAM900102  254 PRAM900103  255 PRAM900104  256 PTIO830101  257 PTIO830102  258 QIAN880101  259 QIAN880102  260 QIAN880103  261 QIAN880104  262 QIAN880105  263 QIAN880106  264 QIAN880107  265 QIAN880108  266 QIAN880109  267 QIAN880110  268 QIAN880111  269 QIAN880112  270 QIAN880113  271 QIAN880114  272 QIAN880115  273 QIAN880116  274 QIAN880117  275 QIAN880118  276 QIAN880119  277 QIAN880120  278 QIAN880121  279 QIAN880122  280 QIAN880123  281 QIAN880124  282 QIAN880125  283 QIAN880126  284 QIAN880127  285 QIAN880128  286 QIAN880129  287 QIAN880130  288 QIAN880131  289 QIAN880132  290 QIAN880133  291 QIAN880134  292 QIAN880135  293 QIAN880136  294 QIAN880137  295 QIAN880138  296 QIAN880139  297 RACS770101  298 RACS770102  299 RACS770103  300 RACS820101  301 RACS820102  302 RACS820103  303 RACS820104  304 RACS820105  305 RACS820106  306 RACS820107  307 RACS820108  308 RACS820109 | AAindex  ID | Feature  ID | AAindex  ID | Feature ID | AAindex  ID | Feature ID | AAindex  ID | Feature ID | AAindex  ID |
| --- | --- | --- | --- | --- | --- | --- | --- | --- | --- |

| Feature  ID  309 RACS820110  310 RACS820111  311 RACS820112  312 RACS820113  313 RACS820114  314 RADA880101  315 RADA880102  316 RADA880103  317 RADA880104  318 RADA880105  319 RADA880106  320 RADA880107  321 RADA880108  322 RICJ880101  323 RICJ880102  324 RICJ880103  325 RICJ880104  326 RICJ880105  327 RICJ880106  328 RICJ880107  329 RICJ880108  330 RICJ880109  331 RICJ880110  332 RICJ880111  333 RICJ880112  334 RICJ880113  335 RICJ880114  336 RICJ880115  337 RICJ880116  338 RICJ880117  339 ROBB760101  340 ROBB760102  341 ROBB760103  342 ROBB760104  343 ROBB760105  344 ROBB760106  345 ROBB760107  346 ROBB760108  347 ROBB760109  348 ROBB760110  349 ROBB760111  350 ROBB760112  351 ROBB760113  352 ROBB790101  353 ROSG850101  354 ROSG850102  355 ROSM880101  356 ROSM880102  357 ROSM880103  358 SIMZ760101  359 SNEP660101  360 SNEP660102  361 SNEP660103  362 SNEP660104  363 SUEM840101  364 SUEM840102  365 SWER830101  366 TANS770101  367 TANS770102  368 TANS770103  369 TANS770104  370 TANS770105  371 TANS770106  372 TANS770107  373 TANS770108  374 TANS770109  375 TANS770110  376 VASM830101  377 VASM830102  378 VASM830103  379 VELV850101  380 VENT840101  381 VHEG790101  382 WARP780101  383 WEBA780101  384 WERD780101  385 WERD780102  386 WERD780103  387 WERD780104  388 WOEC730101  389 WOLR810101  390 WOLS870101  391 WOLS870102  392 WOLS870103  393 YUTK870101  394 YUTK870102  395 YUTK870103  396 YUTK870104  397 ZASB820101  398 ZIMJ680101  399 ZIMJ680102  400 ZIMJ680103  401 ZIMJ680104  402 ZIMJ680105  403 AURR980101  404 AURR980102  405 AURR980103  406 AURR980104  407 AURR980105  408 AURR980106  409 AURR980107  410 AURR980108  411 AURR980109  412 AURR980110  413 AURR980111  414 AURR980112  415 AURR980113  416 AURR980114  417 AURR980115  418 AURR980116  419 AURR980117  420 AURR980118  421 AURR980119  422 AURR980120  423 ONEK900101  424 ONEK900102  425 VINM940101  426 VINM940102  427 VINM940103  428 VINM940104  429 MUNV940101  430 MUNV940102  431 MUNV940103  432 MUNV940104  433 MUNV940105  434 WIMW960101  435 KIMC930101  436 MONM990101  437 BLAM930101  438 PARS000101  439 PARS000102  440 KUMS000101  441 KUMS000102  442 KUMS000103  443 KUMS000104  444 TAKK010101  445 FODM020101  446 NADH010101  447 NADH010102  448 NADH010103  449 NADH010104  450 NADH010105  451 NADH010106  452 NADH010107  453 MONM990201  454 KOEP990101  455 KOEP990102  456 CEDJ970101  457 CEDJ970102  458 CEDJ970103  459 CEDJ970104  460 CEDJ970105  461 FUKS010101  462 FUKS010102  463 FUKS010103  464 FUKS010104  465 FUKS010105  466 FUKS010106  467 FUKS010107  468 FUKS010108  469 FUKS010109  470 FUKS010110  471 FUKS010111  472 FUKS010112  473 MITS020101  474 TSAJ990101  475 TSAJ990102  476 COSI940101  477 PONP930101  478 WILM950101  479 WILM950102  480 WILM950103  481 WILM950104  482 KUHL950101  483 GUOD860101  484 JURD980101  485 BASU050101  486 BASU050102  487 BASU050103  488 SUYM030101  489 PUNT030101  490 PUNT030102  491 GEOR030101  492 GEOR030102  493 GEOR030103  494 GEOR030104  495 GEOR030105  496 GEOR030106  497 GEOR030107  498 GEOR030108  499 GEOR030109  500 ZHOH040101  501 ZHOH040102  502 ZHOH040103  503 BAEK050101  504 HARY940101  505 PONJ960101  506 DIGM050101  507 WOLR790101  508 OLSK800101  509 KIDA850101  510 GUYH850102  511 GUYH850104  512 GUYH850105  513 JACR890101  514 COWR900101  515 BLAS910101  516 CASG920101  517 CORJ870101  518 CORJ870102  519 CORJ870103  520 CORJ870104  521 CORJ870105  522 CORJ870106  523 CORJ870107  524 CORJ870108  525 MIYS990101  526 MIYS990102  527 MIYS990103  528 MIYS990104  529 MIYS990105  530 ENGD860101  531 FASG890101 | AAindex  ID | Feature  ID | AAindex  ID | Feature ID | AAindex  ID | Feature ID | AAindex  ID | Feature ID | AAindex  ID |
| --- | --- | --- | --- | --- | --- | --- | --- | --- | --- |
